# Role of the Prostaglandin E_2_ EP1 Receptor in Traumatic Brain Injury

**DOI:** 10.1371/journal.pone.0113689

**Published:** 2014-11-26

**Authors:** Alexander V. Glushakov, Jawad A. Fazal, Shuh Narumiya, Sylvain Doré

**Affiliations:** 1 Department of Anesthesiology, University of Florida College of Medicine, Gainesville, Florida, United States of America; 2 Department of Pharmacology, Kyoto University Faculty of Medicine, Kyoto, Japan; 3 Departments of Neuroscience, Neurology, Psychiatry and Center for Translational Research in Neurodegenerative Disease, University of Florida College of Medicine, Gainesville, Florida, United States of America; Universidade Federal do Rio de Janeiro, Brazil

## Abstract

Brain injuries promote upregulation of so-called proinflammatory prostaglandins, notably prostaglandin E_2_ (PGE_2_), leading to overactivation of a class of its cognate G-protein-coupled receptors, including EP1, which is considered a promising target for treatment of ischemic stroke. However, the role of the EP1 receptor is complex and depends on the type of brain injury. This study is focused on the investigation of the role of the EP1 receptor in a controlled cortical impact (CCI) model, a preclinical model of traumatic brain injury (TBI). The therapeutic effects of post-treatments with a widely studied EP1 receptor antagonist, SC-51089, were examined in wildtype and EP1 receptor knockout C57BL/6 mice. Neurological deficit scores (NDS) were assessed 24 and 48 h following CCI or sham surgery, and brain immunohistochemical pathology was assessed 48 h after surgery. In wildtype mice, CCI resulted in an obvious cortical lesion and localized hippocampal edema with an associated significant increase in NDS compared to sham-operated animals. Post-treatments with the selective EP1 receptor antagonist SC-51089 or genetic knockout of EP1 receptor had no significant effects on cortical lesions and hippocampal swelling or on the NDS 24 and 48 h after CCI. Immunohistochemistry studies revealed CCI-induced gliosis and microglial activation in selected ipsilateral brain regions that were not affected by SC-51089 or in the EP1 receptor-deleted mice. This study provides further clarification on the respective contribution of the EP1 receptor in TBI and suggests that, under this experimental paradigm, the EP1 receptor would have limited effects in modulating acute neurological and anatomical pathologies following contusive brain trauma. Findings from this protocol, in combination with previous studies demonstrating differential roles of EP1 receptor in ischemic, neurotoxic, and hemorrhagic conditions, provide scientific background and further clarification of potential therapeutic application of prospective prostaglandin G-protein-coupled receptor drugs in the clinic for treatment of TBI and other acute brain injuries.

## Introduction

Traumatic brain injury (TBI) is the deadliest and most disabling form of acute brain trauma and has no current effective treatment. TBI is a complex disorder resulting from coexisting primary and secondary mechanisms such as mechanical brain damage, parenchymal subarachnoid hemorrhages, excitotoxicity, brain edema, and activation of neuroinflammatory pathways [1], [2]. Thus, anti-inflammatory treatment is currently considered one of the promising strategies for TBI [3]. Neuroinflammation involving upregulation of cyclooxygenase (COX) enzymes, primarily inducible COX-2, and subsequent increase in synthesis of different classes of proinflammatory prostaglandins, such as PGE_2_ and PGF_2α_, plays a significant role in the etiopathology of many neurological disorders, including ischemic stroke, epilepsy, and TBI [4]–[13]. Selective and non-selective COX-2 inhibitors have been widely used in the clinic for the treatment of different disorders and preclinical data suggest that their use might also be beneficial in some neurological disorders, including certain types of stroke and TBI [5], [6], [8], [14], [15]. However, the neurological application of COX-2 inhibitors is limited due to serious cerebrovascular, cardiac, and gastrointestinal adverse effects [16]; thus, drugs targeting the downstream effectors of COX-2 cascades, including cognate prostaglandin receptors, have been suggested as a more specific and safe alternative to selective and non-selective COX-2 inhibitors [11], [12]. Various biological actions of the specific prostaglandins are mediated via activation of several different isotypes of their cognate membrane G-protein-coupled receptors (GPCRs), and thus far the data suggest that the prostaglandin receptors, which exert most of their action through activation of intracellular calcium (Ca^2+^)-signaling, such as closely related PGE_2_ receptor EP1 and PGF_2α_ receptor FP [11], [17]–[19], exacerbate neuronal dysfunction after ischemic and excitotoxic brain injuries [11], [17], [19]–[23]. Previous data indicates that genetic deletion or pharmacological blockade of functionally related FP receptor with Ca^2+^-signaling mechanisms [17], [18] is beneficial in stroke [17], [20] and TBI [21], which are consistent with the generally recognized notion that overactivation of the FP receptor in a disease state, with a few exceptions, is deleterious [10]. Nevertheless, based on our recent data obtained using a model of intracerebral hemorrhage (ICH) [24], the roles of prostaglandin receptors are complex and the outcomes of inhibition or genetic deletion of some of these receptors, such as EP1, may have opposing effects in different neurological conditions, such as ischemic and hemorrhagic strokes [19], [22]–[24]. In addition, data obtained in a model of surgical brain injury demonstrated lack of effects of the EP1 receptor inhibitor SC-51089 on edema and cell death [25]. However, in the later study, improvements in neurological deficits were observed at an acute time point, suggesting complexity of the EP1 receptor pathways in models involving mechanical brain injury.

Our previous study concerning the role of the FP receptor in TBI, which is structurally and functionally related to the EP1 receptor, using pharmacological tools and genetic deletion of this receptor (i.e., FP^−/−^ mice) in a preclinical controlled cortical impact (CCI) model of TBI, demonstrated that a single treatment with its selective antagonist administered after experimental TBI would be beneficial in reducing acute neurological deficits, hippocampal edema, and inflammatory reactions such as gliosis and secondary microglial activation. With the known molecular mechanisms of EP1 and FP receptors in intracellular Ca^2+^-signaling [17]–[19], [23], we hypothesize that the EP1 receptor might be involved in similar pathways. CCI is one of the commonly used TBI models, primarily due to the tight control of injury parameters and the resemblance of several anatomical and neurological outcomes with those observed in humans following head trauma [26]–[30]. The CCI model, which is often referred to as a contusion model, was selected due to its distinctive features such as cortical deformation axonal-involving damage of varied degrees not presented in experimental models previously used to study the role of EP1 receptor in acute brain injuries (i.e., transient ischemia, N-methyl-D-aspartate-induced excitotoxicity, ICH, or surgical brain injury). EP1 receptor antagonists have long been suggested as an alternative therapeutic strategy for different neurological conditions, including acute brain injuries and neurodegenerative disorders [11], [12]. However, based on current preclinical data demonstrating the complexity of pathophysiological roles inflowing this receptor in different neurological conditions such as ischemia [19], [22], [31]–[33], hemorrhagic stroke [24], excitotoxicity [19], [22], [32], and surgical brain injury [25], which may affect overall outcomes of pharmacological interventions targeting the EP1 receptor pathways, the goal of this study was to investigate the EP1 receptor as a putative target for development of a novel therapeutic strategy for management of acute TBI to prevent its devastating sequelae by using a comprehensive pharmacological approach, including selective the EP1 receptor antagonist and genetically modified mice lacking the EP1 receptor (EP1^−/−^).

## Materials and Methods

### Ethics Statement

Age-matched adult (2–4 months) wildtype (WT) and EP1^−/−^ C57BL/6 male mice were used in the study, which was carried out in accordance with the recommendations in the Guide for the Care and Use of Laboratory Animals of the National Institutes of Health. All procedures used in this study were approved by the University of Florida Institutional Animal Care and Use Committee. All mice were maintained and housed under controlled conditions (23°C ±2°C; 12-h reversed light/dark cycle), with access to food and water ad libitum. All surgery was performed under isoflurane anesthesia, and all efforts were made to minimize the pain and distress of the experimental animals.

### Experimental Animals and CCI Procedures

All mice used in the study were obtained from the in-house breeding colony and the WT and EP1^−/−^ C57BL/6 genetic backgrounds. Physiological parameters (i.e., mean arterial blood pressure, pH, blood gases PaO_2_ and PaCO_2_, and body temperature), cerebral vessel diameters, and cerebral arterial vasculature morphology are not significantly different between EP1^−/−^ and WT mice [34]. In this study, we used the same CCI or sham procedures as previously described [21]. Briefly, under 2% isoflurane anesthesia, CCI was stereotaxically induced using PCI3000 PinPoint Precision Cortical Impactor (Hatteras Instruments, Cary, NC, USA) with the following parameters: 3 mm diameter impact tip, 3 m/s velocity, 100 ms compression time, and 1 mm compression distance, allowing us to create a reproducible experimental contusive TBI model with mild-to-moderate severity [35]. Sham mice underwent the same anesthesia and craniotomy surgery procedures but not CCI. After the surgery, to prevent dehydration, the mice received an intraperitoneal injection of warm saline and were allowed to recover in a temperature-controlled chamber for at least 1 h before being transferred back to the animal housing.

### Drug Treatments

Pharmacological treatments with the selective EP1 receptor antagonist SC-51089 were performed with minor modifications [19], [23]. Control groups received injections of corresponding vehicle with the same volume and treatment regimen. To study the effect of the EP1 antagonist, WT mice were post-treated with repeated subcutaneous SC-51089 injections at the same doses of 100 µg/kg using a treatment regimen with the maximal effective dose previously reported to improve anatomical and behavioral outcomes following ischemia and excitotoxicity [19], [23]. In these mice, SC-51089 was administered using two injections: the first immediately after CCI and the second after 24 h. In addition, a separate smaller group of animals was treated with 100 µg/kg SC-51089 at three acute time points: 0, 1, and 3 h after CCI. Before repeated injections, mice were briefly anesthetized with isoflurane to avoid potential injury to the surgery site due to handling. Solutions for SC-51089 injections were prepared in sterile saline immediately before use from stock solutions with a concentration of 10 mg/mL in dimethyl sulfoxide that were previously aliquoted and stored at −20°C.

### Neurological Deficit Scores (NDS)

Neurobehavioral deficits were assessed using a 24-point NDS scale in all mice 24 and 48 h after CCI or sham surgeries, as we described in detail elsewhere [21]. NDS was reported as a sum score obtained from the assessment of six individual tests scored between 0 and 4 points for normal performance and according to graduate criteria of severity. The assessment comprised tests for body symmetry, gait, circling behavior, climbing, front limb symmetry, and compulsory circling.

### Stereological Brain Histopathology and Immunohistochemistry

All subjects were survived for 48 h (2 days) post-injury and were then sacrificed. A series of eight 30-µm-thick coronal sections were obtained throughout the entire brain and were processed for histological analysis as described previously [21]. Briefly, to quantitate brain pathology (i.e., lesion volume and hippocampal edema), cresyl violet staining was used. Brain gliosis was assessed by immunohistochemistry for ionized calcium-binding adapter molecule 1 (Iba1) (microglia) and glial fibrillary acidic protein (GFAP) (astrocytes). All slides were scanned using ScanScope (Aperio Technologies, Vista, CA, USA) and analyzed using ImageScope software (Aperio Technologies) in a blinded manner.

### Statistical Analysis

For quantitative histopathological and immunohistochemical data, Student’s t-test was used to compare the drug-treated group with the matching vehicle-treated control group or between genotypes. Statistical comparisons among multiple groups were done using one-way ANOVA. For non-parametric data (i.e., NDS), the Mann-Whitney rank sum test was used. Data are presented as the mean ± standard error, and P values <0.05 were considered significant [36].

## Results

### Anatomical and Behavioral Characteristics of CCI Injury

Based on the previous data indicating that blockade or ablation of Ca^2+^-modulating EP1 receptor [18], [19] provides neuroprotection in ischemic stroke and excitotoxicity models [19], [22], [23], and that the same interventions against the closely related FP receptor with similar mechanisms of action [17] limit anatomical brain damage and neurological deficits following both experimental ischemic stroke [17], [20] and TBI [21], we hypothesized that pharmacological inhibition or genetic deletion of the EP1 receptor may also have a protective effect in a model of TBI. To determine whether EP1 receptor inhibition with a selective antagonist of EP1 receptor SC-51089 or its genetic deletion could provide neuroprotection following acute experimental TBI similarly as it was demonstrated in different models of ischemia and excitotoxicity, the anatomical and neurobehavioral outcomes were compared in WT and EP1^−/−^ mice in separate cohorts. At the 48-h time point, CCI produced consistently significant cortical lesions characterized by morphological alternations in cellular morphology and density, including cell and tissue loss (i.e., cavitation) and parenchymal ICH, whereas in sham animals, only marginal changes were observed in the cortical tissue due to craniotomy surgery. The significant hippocampal swelling (edema) previously reported in this model [21] was also observed in CCI but not in sham animals. Increased NDS was observed in all CCI-injured animals and the NDS values were significantly different from those of sham-injured animals.

### Effects of the Selective EP1 Receptor Antagonist SC-51089 on the Anatomical and Neurobehavioral Outcomes following CCI

**Figure 1:**
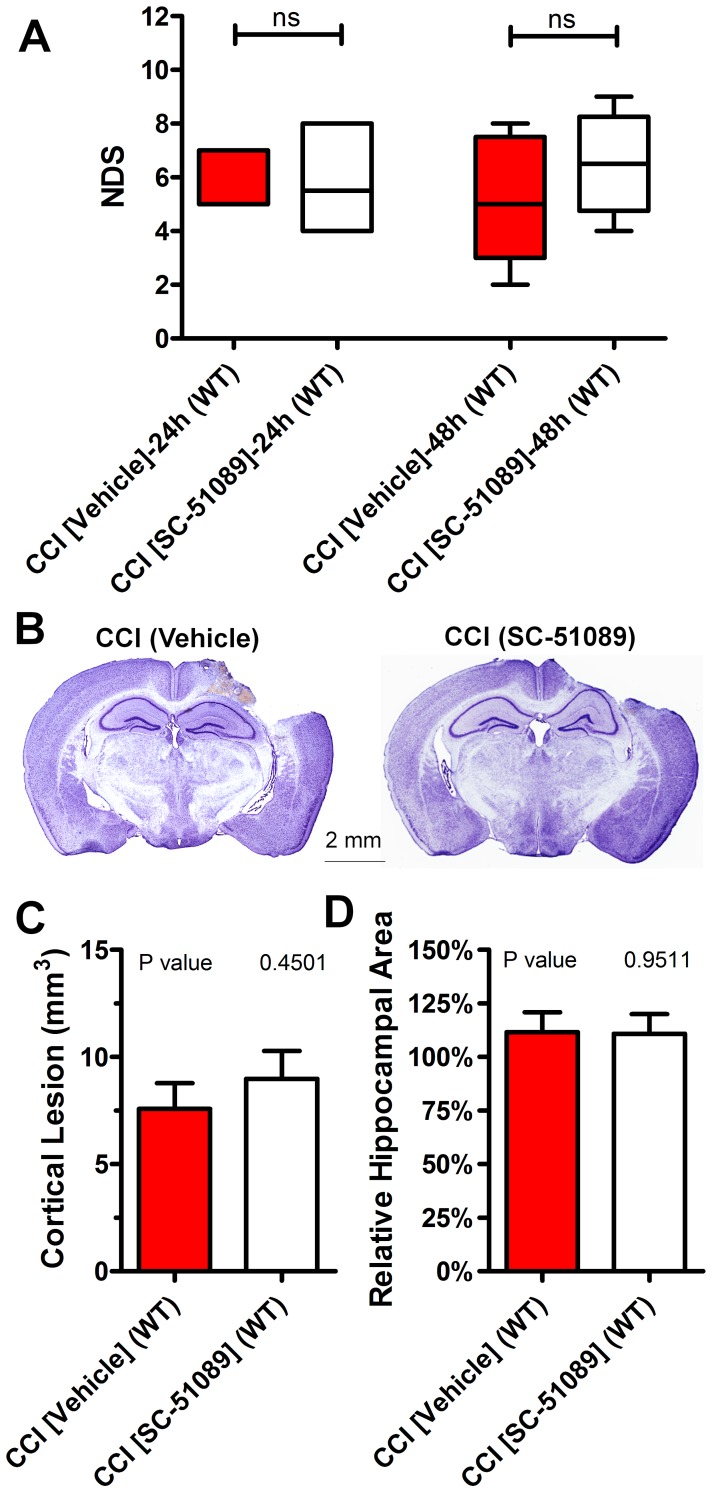
Lack of effect of SC-51089 on brain pathology and NDS after CCI in WT mice. (A) NDS 24 and 48 h assessed for CCI-injured WT mice following vehicle or SC-51089 treatment. (B) Representative cresyl violet-stained brain sections from animals of the same groups assessed 48 h after experimental injury. (C and D) Stereological quantification of the cortical lesion volume (C) and hippocampal edema in the ipsilateral hemisphere expressed as the averaged relative hippocampal area between bregma 1 and 2 normalized to respective values measured on the contralateral side (D) in vehicle and SC-51089-treated animals 48 h after CCI. The “ns” denotes not statistically significant (*P>*0.05, Mann-Whitney rank sum test), *P* values in C and D obtained from Student’s t-test, n = 6–9 per group.

In the first set of experiments, we tested whether post-treatment with SC-51089 could limit anatomical deficits after CCI in WT mice using a dose and treatment regimen reported to improve outcomes in a mouse model of ischemia induced by transient middle cerebral artery occlusion [19]. The analyses of cortical lesion volumes and hippocampal swelling demonstrated that these values 48 h following CCI were not significantly different between vehicle and 100 µg/kg SC-51089- (immediately after CCI and at 24 h) treated mice (Figure 1, A and B). NDS 24 and 48 h after CCI were also not altered following post-treatment with SC-51089 compared to vehicle-treated WT mice (Figure 1C). To further begin addressing a possible relatively short lifetime of SC-51089 and that the therapeutic window in a TBI model for the treatment could be different from the models of ischemia and chemically induced neurotoxicity, we performed a sub-study (n = 4) with acute repetitive treatment involving three 100 µg/kg injections at 0, 1, and 3 h after CCI and measured the same outcomes. The lesion volume at 48 h after CCI in the group of animals with acute repetitive treatment was 6.85±0.65 mm^3^ and the median NDS values were 5.5 (ranged between 3 and 8) and 6 (ranged between 3 and 8) at 24 and 48 h after CCI, respectively. These results revealed no significant differences between lesion volumes in the groups with different SC-51089 treatment regimens and vehicle (P = 0.4791, one-way ANOVA).

### Effects of the EP1 Receptor Knockout on Anatomical and Neurobehavioral Outcomes following CCI

**Figure 2:**
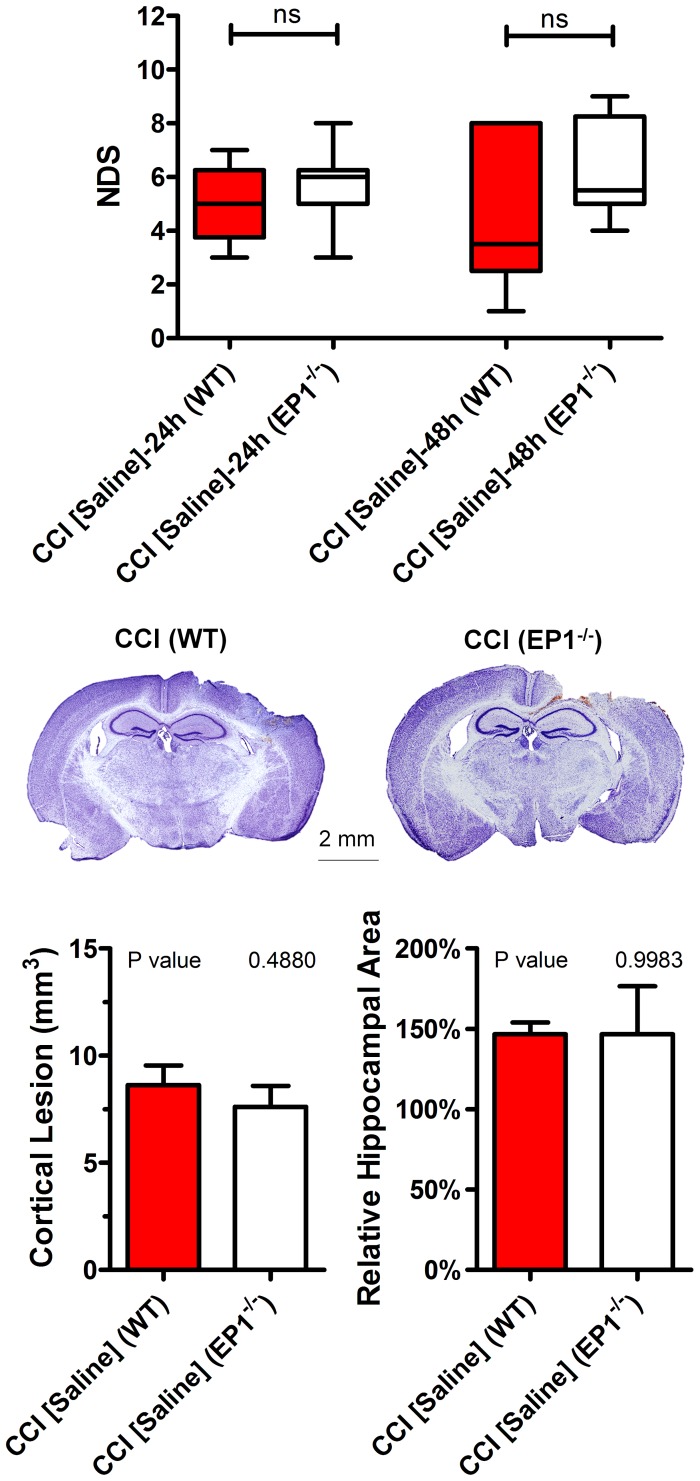
Comparison of brain pathology and neurobehavioral outcomes after CCI in WT and EP1^−/−^ mice. (A) NDS 24 and 48 h in the CCI-injured WT and EP^−/−^ mice. (B) Representative cresyl violet-stained brain sections from animals of the same groups assessed 48 h after experimental injury. (C and D) Stereological quantification of the cortical lesion volume (C) and hippocampal edema in the ipsilateral hemisphere expressed as averaged relative to the hippocampal area between bregma 1 and 2 normalized to respective values measured on the contralateral side (D) 48 h after CCI. N = 6 to 7 per group. The “ns” denotes not statistically significant (*P*>0.05, Mann-Whitney rank sum test), P values in C and D obtained from Student’s t-test, n = 6–8 per group.

Based on the published data that genetic deletion of the EP1 receptor improved anatomical and behavioral outcomes following ischemia and neurotoxicity and to address possible differential bioavailability of the EP1 antagonist in brain structures affected by experimental TBI compared to other models, outcomes following the same CCI or sham procedures were accessed in EP1^−/−^ mice. The CCI procedures performed with the same parameters resulted in similar lesion size and hippocampal swelling with values not significantly different compared to WT mice from the corresponding control group. Similar to WT mice, no considerably significant edema or cortical alternations were observed in EP1^−/−^ mice 48 h following sham procedures (Figure 2, A and B). NDS scores in WT and EP1^−/−^ mice were significantly higher than in the corresponding sham-operated mice 24 and 48 h after procedures, but there were no significant differences in NDS between either time points or between genotypes (Figure 2C).

### Lack of The Effects of the EP1 Receptor Antagonist, and of the EP1 Receptor Knockout, on Microglial Activation and Astrogliosis following CCI

**Figure 3:**
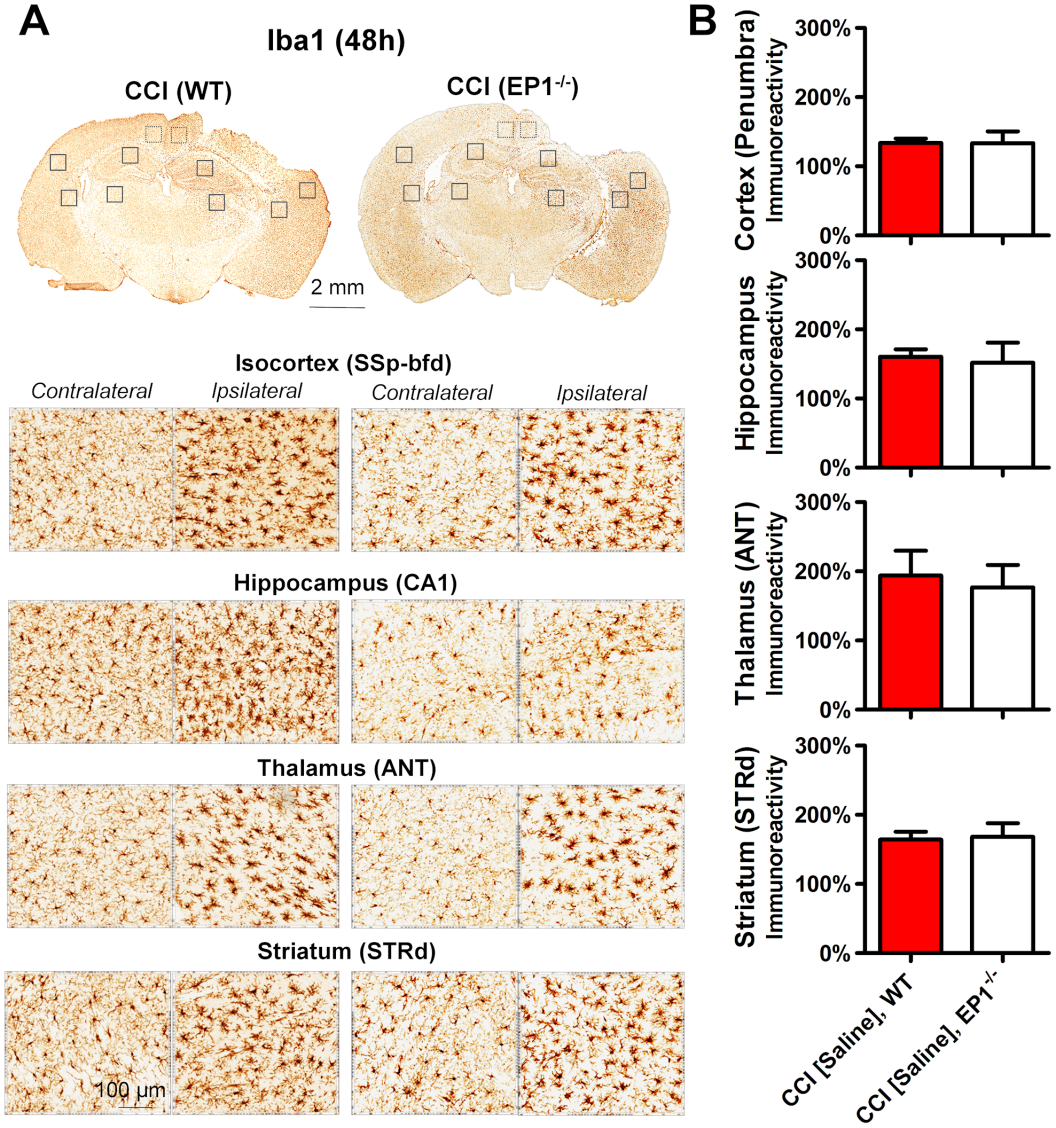
Comparison of Iba1 expression after CCI in WT and EP1^−/−^. (A) Representative brain sections and microphotographs of selected brain regions from the same corresponding sections used for immunohistochemistry quantification. (B) Quantitative analyses of Iba1 immunoreactivity in the ipsilateral brain regions represented as a percentage of their contralateral counterparts showing the lack of the effect of the EP1 knockout compared to WT mice. Data for animals of both genotypes with vehicle and saline treatments were pulled together. The brain regions used in the analyses include (isocortex RSPv) cortical penumbral regions [combined ventral part of retrosplenial area (microphotograph is not shown, but the area is denoted with dotted line in A) and the primary somatosensory area of isocortex (SSp-bfd)], hippocampus (CA1 region), the lateral group of the dorsal thalamus (ANT), and the dorsal region of striatum (STRd). No statistical differences were observed when using Student’s t-test (n = 16 for WT and n = 8 for EP1^−/−^ mice).

**Figure 4:**
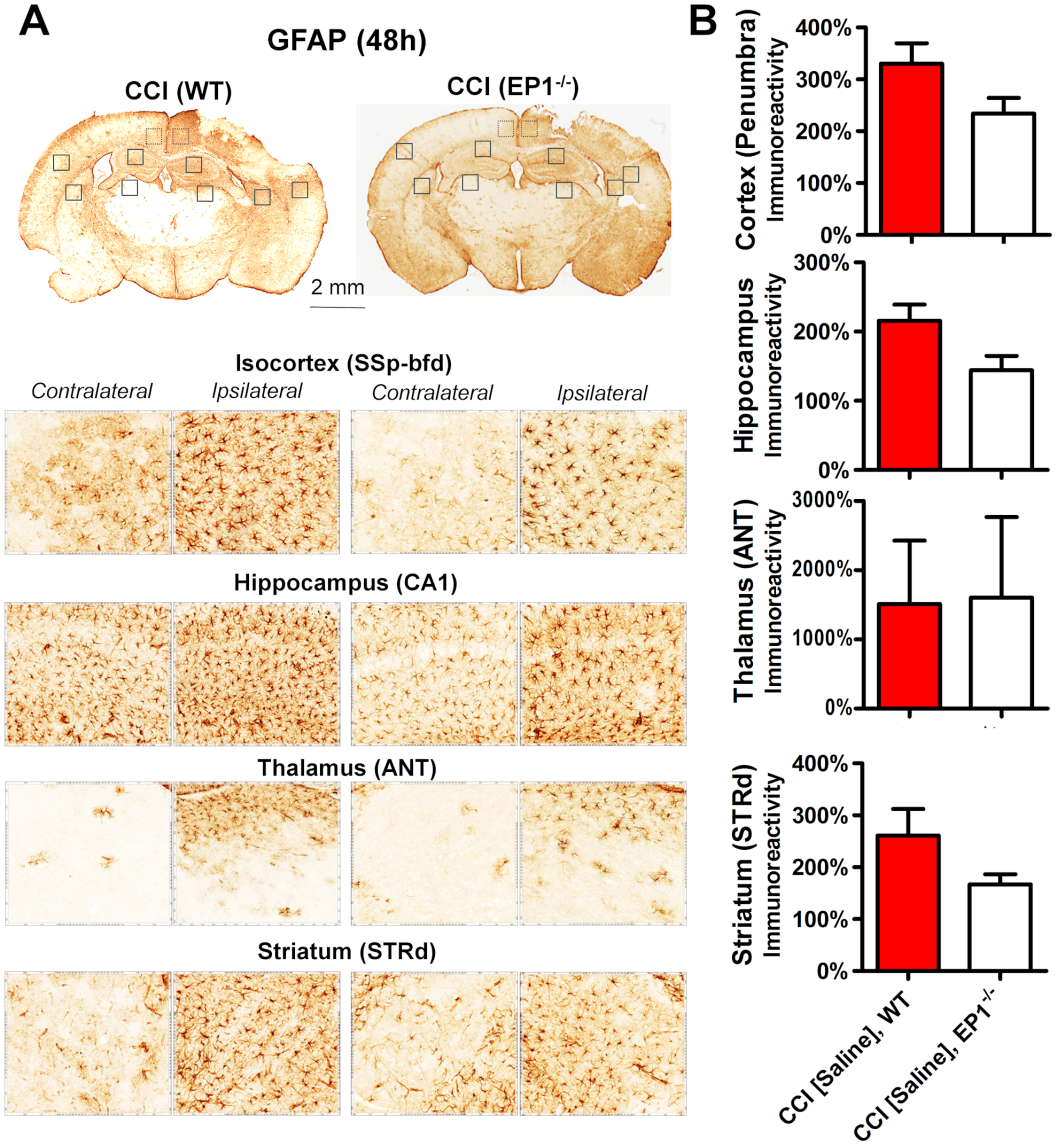
Comparison of GFAP expression after CCI in WT and EP1^−/−^ mice. (A) Representative brain sections and microphotographs of selected brain regions from the same corresponding sections used for immunohistochemistry quantification. (B) Quantitative analyses of Iba1 immunoreactivity in the ipsilateral brain regions represented as a percentage of their contralateral counterparts showing the lack of the effect of the EP1 knockout compared to WT mice. Data for animals of both genotypes with vehicle and saline treatments were pulled together. Description of the brain regions is the same as that in Figure 3. No statistical differences were observed when using Student’s t-test (n = 16 for WT and n = 8 for EP1^−/−^ mice).

Although involvement of reactive microglia and astrocytes causing gliosis in TBI is well established fact [37], their role in the progression of secondary injury is not well understood. Thus, we performed immunohistochemical analyses of adjacent brain sections used for stereological analyses on brain pathologies. The data summarized in Figures 3 and 4 demonstrate that CCI produced increases in Iba1 and GFAP immunoreactivities and changes in cellular morphology in selected brain regions of the ipsilateral hemisphere compared with respective regions of the contralateral side. In the ipsilateral hemisphere, the immunohistochemical alterations in Iba1 and GFAP expressions were observed throughout the cerebral cortex, including the most distal parts of the isocortex, such as the olfactory areas, with the most prominent changes within the site of direct CCI injury, as well as in the surrounding areas often referred to as penumbral regions. The core of CCI injury was located in the primary somatosensory area of the isocortex and extended to its posterior parietal association area and the dorsal part of retrosplenial area. The cortical penumbral regions were observed within both rostral-caudal and dorsal-ventral locations from the injury site. The most affected cortical regions included the retrosplenial, the ventral part of the somatosensory and dorsal auditory areas. In addition, the prominently increased immunoreactivities and morphological alternations of Iba1- and GFAP-immunopositive cells were observed in the selected brain regions not directly affected by CCI injury, including the hippocampal formation, the dorsal areas of striatum, and selected thalamic regions such as the anterior and geniculate groups of thalamus. The data presented in Figures 3 and 4 represent immunohistochemical changes in the brain regions located between the posterior bregma 1 and 2 mm in WT and EP1^−/−^ mice. The immunoreactivity levels in the cortical penumbra were calculated as combined values measured in the ventral part of the retrosplenial area and the primary somatosensory area proximal to the injury site, and immunoreactivity in the thalamus was measured in the area covering the dorsal and lateral nuclei. Although we observed some trends to decrease GFAP immunoreactivity in selected brain regions, these changes were not statistically significant (Figure 4). No significant changes in Iba1 and GFAP expression and no obvious differences in immunopositive cellular morphology were observed between WT and EP1^−/−^ mice and, similarly, no differences were observed between vehicle and EP1 receptor antagonist treatment in WT mice. Thus, these data suggest that the reactive microglial activation and gliosis observed after CCI are not directly dependent on activation of the EP1 receptor.

## Discussion

Based on a number of preclinical in vivo and in vitro studies, selective antagonists of the EP1 receptor have been considered as a promising approach for the treatment of ischemic stroke and other neurological conditions involving acute excitotoxicity as an alternative approach to COX inhibitors [12], [19], [22]. Because of encouraging results from preclinical studies from various teams, including ours, effort have been made to investigate the effects of EP1 receptor inhibitors in preclinical models of several other neurological and neurodegenerative conditions, including epilepsy [38], [39], acute surgical brain injury [25], hemorrhagic stroke [24], and Huntington [40] and Alzheimer’s [33] diseases. However, our recent mouse study indicates that the EP1 receptor would be involved in neuroprotective pathways following ICH [24], suggesting that the role of the EP1 receptor in brain injuries is complex. Thus, our goal was to clarify the EP1 receptor role in acute brain trauma, which shares several features with the above-mentioned neurological disorders [41]. To our knowledge, this is the first study of the therapeutic role of the EP1 receptor antagonist in a CCI preclinical in vivo model of TBI resulting from head trauma in C57BL/6 mice. The results of this study indicate that pharmacological inhibition with a selective antagonist or genetic deletion of the EP1 receptor has no significant effects on the acute brain pathology and neurological outcomes in this CCI model, in contrast to differential effects of the interventions of selectively decreasing EP1 receptor activity in preclinical in vivo models of ischemic and hemorrhagic strokes and excitotoxicity. The lack of effects of EP1 receptor antagonist post-treatment or EP1 receptor knockout on the activation of reactive microglia or astrogliosis at acute time points following TBI allows us to speculate that these inflammatory processes are associated with consequences of primary mechanical injury, such as excitotoxicity and its associated oxidative stress, rather than with overactivation of the EP1 receptor due to increases in PGE_2_ levels.

TBI results in acute and chronic neurological deficits associated with brain damage through several mechanisms, including excitotoxicity, ischemia, brain hemorrhage, and subsequent toxicity caused by hemoglobin breakdown products, diffuse edema, and upregulation of proinflammatory mediators [42], [43]. Diffuse axonal injury is a common feature of human TBI and is one of the important pathways associated with various acute and chronic neurological and cognitive deficits [44]. In addition, acute seizures and chronic epileptogenesis are common complications after TBI [42]. Due to the increased risk of acute seizures following TBI and post-traumatic epilepsy, several common antiepileptic drugs are routinely used in clinical practice, although their efficacy in control of post-traumatic seizures and especially in prophylaxis of post-traumatic epilepsy is disputed [45]–[51]. The CCI model used in our study was initially developed as a TBI model reflecting features of closed-head trauma in humans with distinctive characteristics of axonal injury caused by deformation resulting in delayed axonal degeneration due to secondary injury that spreads over time via the white matter tract [26], [52]. Further CCI models were extended to rodent species and currently are one of the most characterized and commonly used preclinical models for studying various secondary consequences of TBI such as gliosis and activation of neuroinflammatory cascades [27]–[30].

Neuroinflammation involving upregulation of primarily inducible COX-2 and increased synthesis of different classes of prostaglandins, such as generally proinflammatory PGE_2_ and PGF_2α_, play a significant role in the etiopathology of many neurological disorders, including TBI [4]–[8]. The role of COX-2 in TBI is well established and preclinical studies indicate that general COX-2 inhibitors could be beneficial for the treatment of this disorder in humans. However, the application of COX inhibitors in clinical practice is limited because of increased risk of serious cerebro- and cardiovascular side effects [16]. In addition, the role of COX-2 in brain injuries is complex [4]–[6] and involves protective pathways; for example, the protective pathways involving other prostaglandin PGE_2_ receptors, EP2 and EP4, in animal models of ischemic stroke [53]–[55]. Thus, downstream effectors of COX-2, such as prostaglandins and their targetable GPCRs, suggest that certain prostaglandin receptor antagonists could be used as an alternative to COX-2 inhibitors [12] to essentially target only proinflammatory downstream COX-2 cascades.

The data suggest that the prostaglandin receptors, which mediate most of their actions through mobilization of intracellular Ca^2+^, such as PGE_2_ receptor EP1 and PGF_2α_ receptor FP [11], [17]–[19], exacerbate neuronal dysfunctions after brain injuries [11], [17], [19]–[22] and promote seizure propagation [38], [39]. However, our most recent data documented that systemic administration of a selective FP receptor antagonist significantly improved anatomical and functional outcomes in various brain injury models, both in vivo and in vitro, including ischemic stroke, excitotoxicity, and TBI, and the selectivity of an FP antagonist was confirmed in genetic knockout mice lacking the FP receptor (FP^−/−^) [17], [19]–[21], [23]. Based on the data from our group and others, the roles of prostaglandin receptors are complex and the outcomes of inhibition or genetic deletion of some of these receptors, such as EP1, may have the opposite effects in different neurological conditions such as ischemic and hemorrhagic strokes [19], [22]–[24]. Our recent data obtained in the preclinical model of ICH using intrastriatal injection of collagenase demonstrated that EP1^−/−^ mice exhibit worsened outcomes of brain lesion volumes and neurological and sensorimotor deficits compared with WT mice, possibly affecting microglial phagocytosis [24].

Our hypothesis of the involvement of the EP1 receptor cascade in the pathologies following TBI was based on the wealth of literature data showing COX-2 upregulation following brain injuries such as stroke and trauma, and the data demonstrating the neuroprotective role of pharmacological and genetic ablation of the EP1 receptor in the in vitro models of excitotoxicity and hemin toxicity [18], [19], [34] in combination with the similar protective effect in the in vivo models of ischemic stroke and excitotoxicity [19], [22], [23], which share several common features with TBI. Moreover, using a mouse model of cerebral ischemia and Alzheimer’s disease, we have shown that EP1 receptor blockade could be beneficial for treatment of both conditions [33]. In addition, recent data from another group indicate that the EP1 receptor antagonist used in our study demonstrated strong a therapeutic effect of repeated treatments with SC-51089 (40 µg/kg) in a murine model of Huntington’s disease and pointed out this compound as a new therapeutic candidate for motor and cognitive deficits characteristic for this and other neurodegenerative disorders [40]. Our previous preclinical data also suggested potential therapeutic applications of the antagonists of prostaglandin F_2α_ FP receptor, which exerts Ca^2+^-modulating effects similar to the EP1 receptor in ischemic stroke and TBI, and these data indicate that despite considerable differences between the mechanisms of primary injury in these models, beneficial effects were obtained with similar treatment regimens [18], [20], [21]. Interestingly, EP1 and FP receptors, in additional to their functional similarity, are also phylogenetically related [11] and preclinical data indicate similar beneficial effects of their pharmacological blockade or genetic deletion in models of ischemia and excitotoxicity [19], [22], [31]. These data also suggest similar therapeutic windows for antagonists from different chemical classes [10]. The experimental data obtained in previous experimental ischemia and excitotoxicity studies using C57 mouse in the Kawano *et. al.* [19] study and revealed the same beneficial effects of EP1 receptor blockade or knockout. Thus, based on the data concerning identified neuroprotective effects of EP1 receptor inhibition and deletion in ischemia and excitotoxicity, we hypothesized that blockade of the EP1 receptor with a selective antagonist would also be beneficial in a preclinical model of TBI, similar to the effects of pharmacological and genetic interventions targeting the closely related FP receptor demonstrating protective effects in all three models. In addition, based on the detrimental effect of EP1 receptor activation in the seizure models [38], [39], blockade of this receptor after TBI could provide additional protection from post-traumatic seizures, which is a common complication following brain injuries.

The main goal of this study was to determine whether pharmacological interventions targeting COX-2 downstream cascades involving the EP1 receptor could be beneficial for the management of TBI and the development of novel therapeutic strategies for its effective treatment. The experimental design of drug treatment was based on the published study performed in models of excitotoxicity and ischemia in the WT and EP1^−/−^ mice [19]. We used the treatment regimen for this compound from an aforementioned study showing beneficial effect, and based on the similarity of the effect of the inhibitor of the related FP receptor in stroke and TBI [17], [20], [21]. The similar relative effect of SC-51089 on behavioral and anatomical outcomes of experimental stroke and excitotoxicity compared to controls [19], [23], [31], [40] was observed in the study with another EP1 receptor antagonist, ONO-8713, in similar experimental conditions [32], and these outcomes were comparable to the outcomes measured in the EP1^−/−^ mice with and without drug treatment, suggesting appropriate SC-51089 bioavailability with the treatment regimen used in this study. The effective doses of SC-51089 in published studies focusing on ischemia and excitotoxicity are between 5 and 100 µg/kg [19], [23], [31], [40] and the data suggest a wide therapeutic window for application of this compound (up to 12 h after permanent or transient focal ischemia) [31], which would be consistent with temporal profiles of COX-2 activation and progression of secondary injuries following ischemia [56], [57] and TBI [5], [6], [8], [14], [58]. However, the published data indicate that the therapeutic effect of SC-51089 mouse amygdala kindling model of temporal lobe epilepsy could be obtained at more than three orders of magnitude higher doses [38]. The data reported here were obtained using repeated treatment with SC-51089 at doses of 100 µg/kg, which was shown to have the most prominent protective effect in ischemia and excitotoxicity [19] using the same considerations and approach as we applied in our previous TBI study [21]. Initially, we tried to use this compound at lower doses (i.e., 20 µg/kg), but we did not observe changes in neurological deficits after treatment (data not shown). To overcome the possibility of nonoptimal timing or low dose due to lack of the pharmacokinetic and bioavailability data, in this study, all TBI outcomes measured in WT mice were compared with those obtained in EP1^−/−^ mice, and all drug treatment experiments performed in WT mice were replicated in EP^−/−^ mice. The lack of effects of the EP1 receptor pharmacological blockade or knockout on either neurobehavioral and anatomical outcomes or Iba1 and GFAP immunohistochemistry in our *in vivo* CCI model of TBI, which is in contrast with the neuroprotective effects observed in the related in vitro studies [18], [34] and the in vivo models of ischemia and excitotoxicity [19], [22], [23], [40], suggests that involvement of other cell types expressing the EP1 receptor contribute considerably to the brain pathology following TBI. Our data is consistent in part with a report demonstrating a lack of the protective effects of pretreatment with EP1 receptor inhibition in a model of blade incisions leading to localized surgical brain extraction followed by electrocautery by Dr. J. H. Zhang’s group [25]. It should be noted that there are substantial differences in mechanisms, in neurological and behavioral outcomes, and in pharmacological treatment strategies between brain surgery and TBI, reflected in the experimental design in respective preclinical studies such prophylactic pretreatment [25] and therapeutic post-treatment with neuroprotective drugs, respectively. In contrast to our study, statistically significant improvements in behavioral outcomes were observed following pretreatment with the EP1 receptor blocker prior surgical brain injury [25]. These differences could be explained by differences between types of injury and treatment regimens [59]. Even taking into account the differences between axonal injury caused by direct surgical resection of brain tissues, including dura matter, in the surgical brain injury model [25] and varied degrees of axonal damage resulting from brain deformation observed in the CCI model, these data suggest that blockade of EP1 receptors is not protective against mechanical axonal injury. On the other hand, we did not observe exacerbated brain pathologies or neurological deficits in EP1^−/−^ mice in contrast to those observed in an ICH model of hemorrhagic stroke [24]. Taken together, the above-mentioned data and the data obtained in this pilot study allow us to speculate that in mild-to-severe TBI, the neuroprotective and neurotoxic pathways involved in the different components of brain trauma might be mutually compensated and may depend on the temporal resolution of the mechanisms involved. Further detailed studies on the temporal profiles of the different EP1 receptor pathways would provide additional information on the utility of the use EP1 receptor modulators in acute brain injuries.

The differential roles of the EP1 receptor in acute brain injuries might be attributed to its well-known vascular effects. The reports indicate that activation of the EP1 receptor is involved in PGE_2_-mediated vasoconstriction and that male EP1^−/−^ mice have significantly reduced blood pressure compared to WT mice [60]. In addition, EP1^−/−^ mice have enhanced cerebral blood flow at reperfusion in a ischemic stroke model [34]. However, there are no significant morphological or anatomical differences in cerebrovascular vasculature between EP1^−/−^ and WT mice or between FP^−/−^ and WT mice in our related studies [21], [34]. Vascular damage plays an important role in TBI and is a primary cause of ICH, which correlates with TBI severity [61] and triggers secondary biochemical cascades, exacerbating primary injury involving several events associated with disrupted Ca^2+^ signaling such as extensive glutamate release and calpain-dependent cortical necrosis [61]–[64].

Neuroinflammatory processes involving activation of reactive microglia and gliosis are well-established hallmarks of TBI and the data show that neuroinflammation following brain injuries is associated with COX-2 expression [8], [65]. In addition to neuronal expression, increased COX-2 expression following injury has been reported in microglial cells and astrocytes. Our previous study demonstrated that inhibition or genetic deletion of the FP receptor is associated with decreased Iba1 and GFAP immunoreactivity following CCI compared with vehicle-treated WT mice. The important finding of the present study is that neither ablation nor activation of EP1 receptor had a significant effect on Iba1 or GFAP expression, suggesting that microglial and astrocytic markers are not directly affected by EP1 receptor activity and are likely associated with other mechanisms of injury, including possible roles of other prostaglandin receptors.

In conclusion, the data obtained in this study extend our understanding of pathophysiological downstream COX-2 pathways in acute brain injuries. Based on the literature data demonstrating that COX-2 upregulation and increased levels of prostaglandin precursor start within hours [5], [6], [8], [14] and remain for several days after brain trauma [58], in combination with our data from this and previous studies demonstrating differential roles of EP1 and FP receptor modulators, we suggest the potential application and limitations of prospective drugs acting on the prostaglandin receptors in clinical practice for the treatment of TBI and other acute brain injuries.
